# The clinical outcome of minor changes in serum creatinine for patients after curative gastrectomy: a prospective study

**DOI:** 10.3389/fonc.2024.1416888

**Published:** 2024-08-21

**Authors:** Wen-Tao Cai, Xiu-Ya Zeng, Yun-Shi Huang, Wei-Sheng Chen, Xiang-Jian Chen, Xian-Hai Xie

**Affiliations:** ^1^ Department of Traumatic Surgery, The First Affiliated Hospital, Wenzhou Medical University, Wenzhou, Zhejiang, China; ^2^ Acupuncture Massage & Physical Therapy, The First Affiliated Hospital, Wenzhou Medical University, Wenzhou, Zhejiang, China; ^3^ Department of Gastrointestinal Surgery, The First Affiliated Hospital, Wenzhou Medical University, Wenzhou, Zhejiang, China

**Keywords:** gastric cancer, serum creatinine changes, postoperative outcomes, nomogram, prospective study

## Abstract

**Introduction:**

Patients with renal insufficiency are more prone to postoperative complications (PCs). Studies have shown that minor changes in serum creatinine (SCr), immediately post-surgery, can aid in assessing patients’ renal function. This study aimed to explore the relationship between the changes in SCr and PCs in patients with gastric cancer (GC).

**Materials and methods:**

We prospectively collected data regarding the SCr of 530 GC patients, within 2 weeks before surgery and within 24 hours after surgery in our hospital (2014–2016). The patients were divided into three groups according to the level of SCr change after surgery: reduced (<10%), normal (10%), and elevated (>10%) creatinine groups. Univariate and multivariate logistic analysis were performed to evaluate its correlation with short-term PCs in the patients. The R language was used to construct a nomogram.

**Results:**

83, 217, and 230 patients were assigned to the elevated, reduced, and normal SCr groups, respectively. Multivariate analysis showed that the reduced and elevated SCr groups were independently associated with the occurrence of PCs and severe postoperative complications (SPCs), respectively. Additionally, postsurgical SCr change, age, hypoalbuminemia, total gastrectomy, combined resection, and laparoscopy, were independently related to PCs. Combining the above influential factors, the predictive model can distinguish patients with PCs more reliably (c-index is 0.715).

**Conclusion:**

Post-surgery, reduced SCr is a protective factor for PCs, while elevated serum creatinine is an independent risk factor for SPCs. Our nomogram can identify GC patients with high risks of PCs.

## Introduction

Gastric cancer (GC) is one of the most commonly diagnosed malignancies and the third leading cause of cancer-related deaths worldwide (1). More than 20% of global new cancer cases and cancer-related deaths occur in China (2). Radical gastrectomy with D2 lymph node dissection is considered the standard treatment for GC but carries a 12.8–14% incidence of postoperative complications (PCs) (3–7). Postoperative complications (PCs) not only increase costs and prolong hospital stays, but also affect subsequent treatment, and may lead to poor long-term prognosis for GC patients (8, 9). Hence, early identification and intervention for high-risk GC are important.

The continuous improvement of medical standards has given patients with chronic health problems, such as renal insufficiency, the opportunity to be successfully treated through surgical operations (10, 11). As an indicator of the risk in perioperative surgery, preoperative renal insufficiency has also been reported to affect the outcome of patients after surgery (12–14). Recent literature states that the measurement of changes in the level of serum creatinine (SCr) immediately after surgery is also related to the patient’s renal function (15, 16). In our clinical work, we have observed that some patients with gastric cancer have different degrees of change in SCr after surgery. In the follow-up clinical diagnosis and treatment process, we found that the clinical prognosis of patients with varying degrees change in SCr, undergoing radical gastric cancer surgery during the same period, is not the same.

Currently, there are no reports on the influence of SCr level changes on the clinical prognosis of GC patients. Thus, the purpose of this study was to explore the correlation between different degrees of postoperative SCr changes and the short-term outcomes of patients after radical gastrectomy.

## Methods

### Study patients

**Figure 1:**
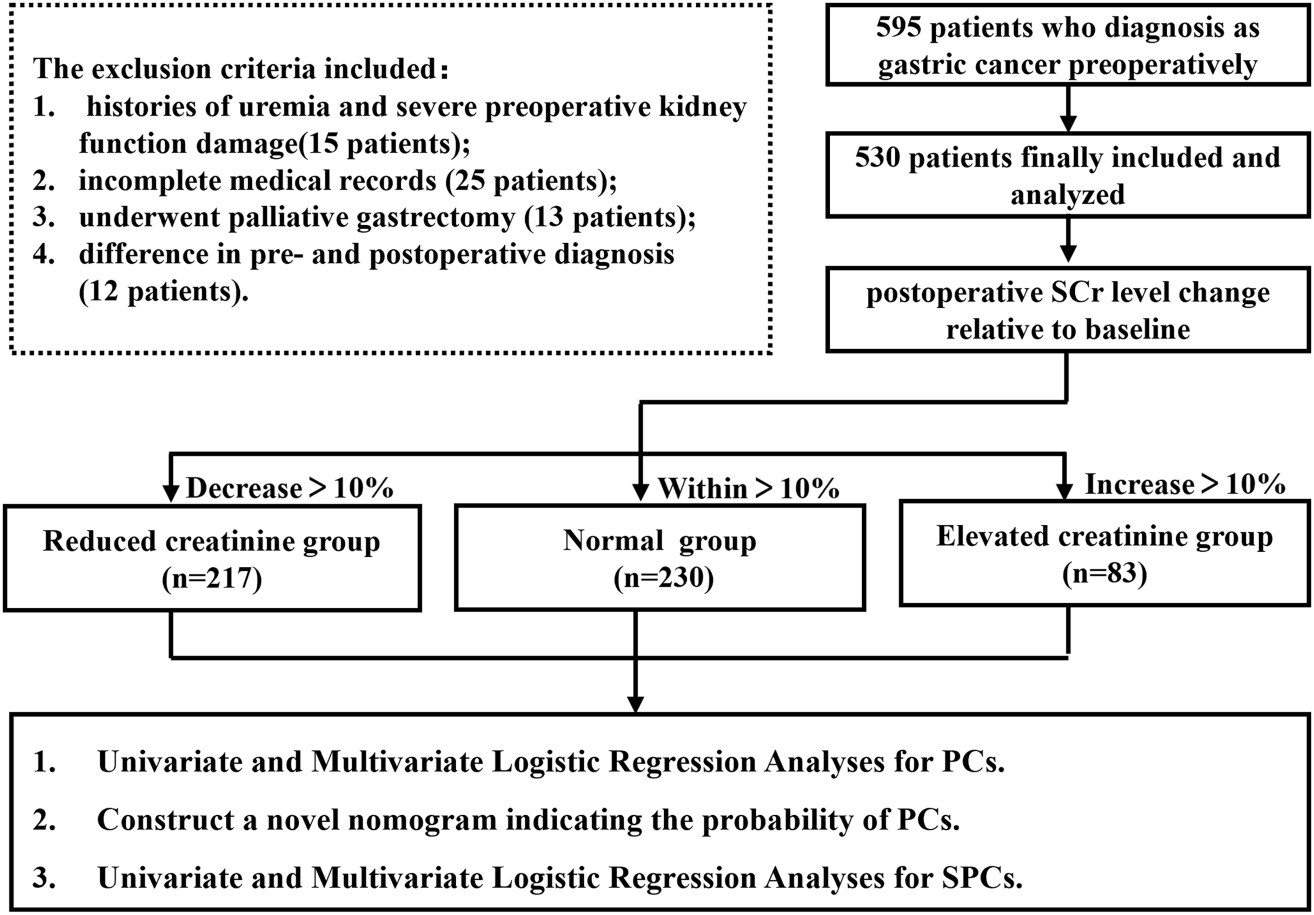
Block flow chart of experimental grouping.

Overall, 595 GC patients were recruited at the First Affiliated Hospital of Wenzhou Medical University from 2014 to 2016. The exclusion criteria included: (1) history of uremia and severe preoperative kidney function damage; (2) history of emergency surgery; (3) incomplete medical records; (4) underwent palliative gastrectomy; or (5) difference in pre- and postoperative diagnosis. After screening, 65 patients were excluded and 530 patients who underwent radical gastrectomy were included and analyzed in this study. The experimental flow chart is shown in **Figure 1**. All eligible participants were requested to provide written informed consent before participating. All GC patients in this study received conventional therapy complies with the Japanese Gastric Cancer Treatment Guidelines (17).

### Data collection

The relevant data were prospectively acquired and analyzed: (1) age; sex; body mass index (BMI); concentration of albumin, hemoglobin, and SCr; neutrophil/lymphocyte ratio (NLR); platelet/lymphocyte ratio (PLR); diabetes mellitus; hypertension; abdominal surgery; comorbidities; American Society of Anesthesiologists (ASA) grade; Nutritional Risk Screening 2002 (NRS 2002) score; tumor location, size, pathological type and tumor-node-metastasis (TNM) stage (preoperative patients and disease characteristics aspect); (2) type of resection, combined resection, surgical method, and type of reconstruction (operative details aspect); and (3) PCs, length of postoperative hospital stay, and hospital costs (postoperative outcomes aspect).

Two researchers recorded the grade of complications within one month after surgery according to the Clavien–Dindo classification (18), and PCs (≥ grade II) were analyzed in this study. Severe postoperative complications (SPCs) were defined as Clavien–Dindo classification ≥ grade III.

### Definition and grouping

The SCr levels were prospectively collected within two weeks before surgery and within 24 hours after surgery in 530 patients who underwent curative gastrectomy in our hospital. All preoperative and postoperative SCr measurements were obtained from the same biochemical laboratory. The primary independent variable of interest was percent change in ΔSCr, calculated as [(postoperative SCr − preoperative SCr)/preoperative SCr] × 100. %ΔSCr was classified into three levels for purposes of analysis: %ΔSCr decrease<10% (defined as the reduced SCr group), %ΔSCr within 10% (defined as the normal SCr group), and %ΔSCr increase >10% (defined as the elevated SCr group).

### Statistical analysis

Categorical variables were expressed in terms of quantity (percentage), while continuous variables conforming to normal distribution are described by the mean (standard deviation); otherwise, they are described by the median (interquartile range). The Kolmogorov–Smirnov test was used to assess the normality of continuous variables. In univariate analysis, Chi-square test and Fisher’s exact test were used to analyze the differences in categorical variables, while the independent t-test and Mann–Whitney U test were used for continuous variables. In the subsequent multivariate analysis, the variables with P <0.10 were included in the univariate analysis into the multivariate logistic regression analysis to calculate the odds ratio (OR) and 95% confidence interval (CI) of the independent variables. A nomogram composed of independent risk factors obtained by multivariate analysis is used to construct a predictive model and calculate the probability of PCs (19).

Values of P < 0.05 were considered statistically significant and all tests were two-tailed. All data analyses were performed using SPSS software (version 22.0; SPSS Inc., Chicago, IL, USA). The prediction model was constructed using the R statistical software (The R Foundation, Vienna, Austria). The generated graphical displays were analyzed using the rms package.

## Results

### Patients

According to the percent change in ΔSCr, the remaining 530 patients were divided into three groups: 230 (43.4%) into the normal SCr group, 83(15.7%) into the elevated SCr group, and 217 (40.9%) into the reduced SCr group. The detailed clinical characteristics of the patients are shown in **Table 1**. There were significant intergroup differences in age (P = 0.004), Charlson comorbidity index (P = 0.017), intraoperative bleeding (P = 0.047), postoperative hospital stays (P = 0.011), and hospitalization expense (P = 0.027). Furthermore, the reduced SCr group had a lower mean hospitalization expense than the normal group and elevated SCr groups (P < 0.05).

**Table 1:** Patient demographic and clinical characteristics.

Factors	Total (n = 530)	Normal group (n=230)	Elevated creatinine group (n=83)	Reduced creatinine group (n=217)	P value
Age, median, years ^*^	65 (58-73)	67 (59-74)	66 (60-74)	64 (56-70)	0.004 ^‡^
BMI, kg/cm2 ^*^	22.2 (20.4-24.2)	22.4 (20.4-24.3)	21.7 (19.8-23.2)	22.5 (20.4-24.4)	0.435
Gender					0.180
Female	129 (24.3)	51 (22.2)	18 (21.7)	60 (27.6)	
Male	401 (75.5)	179 (77.8)	65 (78.3)	157 (72.4)	
Preoperative albumin, g/L^*^	38.5 (35.0-41.5)	38.3 (34.7-41.5)	37.2 (34.2-40.9)	39.1 (36.0-41.8)	0.697
Preoperative hemoglobin, g/L^*^	124 (108-138)	126 (107-139)	120 (100-133)	126 (111-140)	0.593
PLR^*^	144 (112-198)	144 (113-200)	163 (128-253)	124 (110-139)	0.092
NLR ^*^	2.2 (1.7-3.1)	2.3 (1.7-3.1)	2.5 (1.8-3.4)	2.1 (1.6-2.8)	0.068
Hypertension					0.057
No	393 (74.2)	164 (71.3)	57 (68.7)	172 (79.3)	
Yes	137 (25.8)	66 (28.7)	26 (31.3)	45 (20.7)	
Diabetes Mellitus					0.515
No	467 (88.1)	204 (88.7)	66 (79.5)	197 (90.8)	
Yes	63 (11.9)	26 (11.3)	17 (20.5)	20 (9.2)	
CCI					0.017 ^‡^
0	266 (50.2)	104 (45.2)	35 (42.2)	127 (58.5)	
1-2	242 (45.7)	118 (51.2)	42 (50.6)	82 (37.8)	
3-6	22 (4.2)	8 (3.5)	6 (7.2)	8 (3.7)	
ASA score					0.558
1-2	437 (82.5)	187 (81.3)	69 (83.1)	181 (83.4)	
3-4	93 (17.5)	43 (18.7)	14 (16.9)	36 (16.4)	
NRS score					0.318
1-2	332 (62.6)	144 (62.6)	48 (57.8)	140 (64.5)	
3-4	160 (30.2)	67 (29.1)	25 (30.1)	68 (31.3)	
5-6	38 (7.2)	19 (8.3)	10 (12.0)	9 (4.1)	
Intraoperative Bleeding					0.047 ^‡^
No	450 (84.9)	189 (82.2)	68 (81.9)	193 (88.9)	
Yes	80 (15.1)	41 (17.8)	15 (18.1)	24 (11.1)	
Time of Operation, min					0.726
< 210	287 (54.2)	128 (55.7)	46 (55.4)	113 (52.1)	
≥ 210	243 (45.8)	102 (44.3)	37 (44.6)	104 (47.9)	
Intraoperative blood transfusion					0.027 ^‡^
No	496 (93.6)	214 (93.0)	73 (88.0)	209 (96.3)	
Yes	34 (6.4)	16 (7.0)	10 (12.0)	8 (3.7)	
TNM stages					0.128
I	168 (31.7)	67 (29.1)	26 (31.3)	75 (34.6)	
II	115 (21.7)	49 (21.3)	16 (19.3)	50 (23.0)	
III	247 (46.6)	114 (49.6)	41 (49.4)	92 (42.4)	
Postoperative hospital stays, days ^*^	13 (11-18)	14 (11-19)	13 (11-20)	13 (10-16)	0.011 ^‡^
Hospitalization expense, ¥ ^*^	57366 (49674-69926)	59994 (50467-72863)	59558 (51137-75780)	55953 (48023-65539)	0.027 ^‡^

BMI, body mass index; PLR, platelet/lymphocyte ratio; NLR, neutrophil/lymphocyte ratio; CCI, Charlson comorbidity index; ASA, American Society of Anesthesiologists; NRS, nutritional risk screening; TNM, tumor-node-metastasis.

Values are number of patients and percent unless indicated otherwise; *Values are median (inter quartile range). ‡Values are statistically significant (P<0.05).

### The detail of each complication

As seen in **Table 2**, 193 postoperative events occurred in 116 patients (21.9%). Among them, 55 (10.4%) patients had grade IIIa or higher PCs. Abdominal complications (mainly intra-abdominal infections, postoperative bleeding, and bowel obstruction) and pulmonary complications (mainly including pulmonary infections and pleural effusions) were the most frequent postoperative events.

**Table 2:** Actual number and frequency of each complication (Grade ≥ II).

Complication	Total (n=530)	Normal group (n=230)	Elevated creatinine group (n=83)	Reduced creatinine group (n=217)	P values
Wound infection	6 (1.2)	1 (0.4)	1 (1.2)	4 (1.8)	>0.05
Intra-abdominal infection	41 (7.8)	21 (9.1)	6 (7.2)	15 (6.9)	>0.05
Pulmonary	47 (8.9)	22 (9.7)	14 (16.7)	11 (5.1)	<0.01 **^‡^**
Anastomotic leakage	9 (1.7)	6 (2.6)	2 (2.4)	1 (0.5)	>0.05
Thrombosis	17 (3.2)	11 (4.8)	4 (4.8)	2 (0.9)	<0.05 **^‡^**
Bowel obstruction	15 (2.8)	7 (3.0)	3 (3.6)	5 (2.3)	<0.05 **^‡^**
Postoperative bleeding	16 (3.0)	7 (3.0)	5 (6.0)	4 (1.8)	>0.05
Gastroparesis	6 (1.2)	5 (2.2)	0 (0.0)	1 (0.5)	>0.05
Hepatic	3 (0.6)	1 (0.4)	1 (1.2)	1 (0.5)	>0.05
Lymphorrhagia	3 (0.6)	0 (0.0)	0 (0.0)	3 (1.4)	<0.05 **^‡^**
Renal	4 (0.8)	2 (0.9)	2 (2.4)	0 (0.0)	>0.05
Heart	2 (0.4)	1 (0.4)	1 (1.2)	0 (0.0)	>0.05
Hypoalbuminemia	16 (3.0)	8 (3.5)	5 (6.0)	3 (1.4)	>0.05
Others ^*^	8 (1.5)	2 (0.9)	4 (4.8)	2 (0.9)	<0.05 **^‡^**
Death	3 (0.6)	1 (0.4)	1 (1.2)	1 (0.5)	>0.05

Values are number of each complication and percent unless indicated otherwise; *Others contain three severe complication (biliary fistula, Abdominal pseudocyst formation and pancreatic fistula) and five mild complications (intractable hiccup, oral herpes, delirium, skin allergies and gout attack); ‡Values are statistically significant (P<0.05); The chi-square test is used for P values.

**Figure 2:**
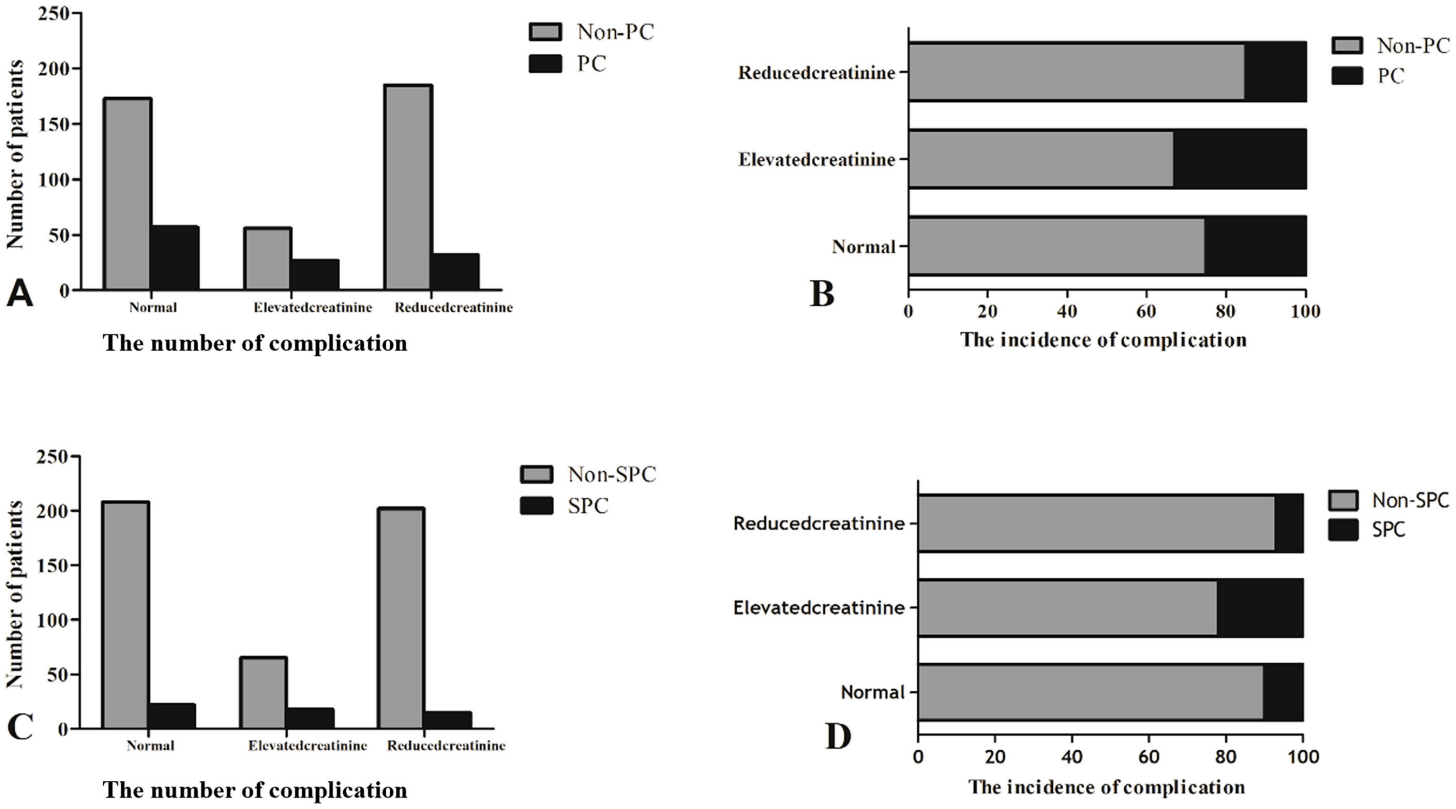
The number of patients with PCs and the incidence of complications between the three groups. **(A, C)** The number of complication. **(B, D)** The incidence of complication.

The number of patients with PCs and the incidence of complications between the three groups are shown in **Figure 2**. Patients with PCs had longer hospital stays (PCs vs. non-PCs: 25 vs. 12 days, P <0.001) and incurred higher hospital costs (PCs vs. non-PCs: 78,781.4 ¥ vs. 54,790.1 ¥, P <0.001) than those who did not.

### Univariate and multivariate analyses logistic regression analyses for PCs

The chi-square test was used to assess the relationship between clinical characteristics of GC patients and the occurrence of PCs. The univariate analysis results showed that age (P < 0.001), NRS score (P = 0.01), hypoalbuminemia (P < 0.001), anemia (P = 0.086), NLR (P = 0.029), Charlson comorbidity index (P = 0.001), ASA score (P = 0.017), Charlson comorbidity index (P < 0.0001), ASA (P = 0.008), tumor size (P = 0.001), TNM stages (P = 0.001), total gastrectomy (P < 0.001), combined resection (P < 0.001), surgical method (P < 0.001), reconstruction methods (P = 0.005), intraoperative bleeding (P = 0.028), and postsurgical SCr change (P = 0.011) differed significantly (**Table 3**). Variables with a P value < 0.1 on univariate analysis were included in the multivariate logistic regression analysis. Age (OR = 1.785, P = 0.012), hypoalbuminemia (OR = 4.506, P = 0.004), total gastrectomy (OR = 1.89 1, P = 0.005), combined resection (OR = 2.231, P = 0.020), laparoscopic surgery (OR = 0.453, P = 0.013), and postsurgical SCr change were independently associated with PCs.

**Table 3:** Univariate and multivariate analysis associated with postoperative complications.

Factors	Univariate Analysis			Multivariate Analysis	
	Non- PC (n=414)	PC (n=116)	P values	OR (95% CI)	P values
Age, years			<0.001^‡^		
< 70	286 (69.1)	56 (48.3)		1	
≥ 70	128 (30.9)	60 (51.7)		1.755 (1.118-2.753)	0.014 ^‡^
Gender			0.954		
Female	101 (24.4)	28 (24.1)			
Male	313 (75.6)	88 (75.9)			
BMI, kg/cm2			0.665		
< 25	339 (81.9)	97 (83.6)			
≥ 25	75 (18.1)	19 (16.4)			
NRS score			0.010 ^‡^		
1-2	267 (64.5)	65 (56.0)			
3-4	125 (30.2)	35 (30.2)			
5-6	22 (5.3)	16 (13.8)			
Hypoalbuminemia, g/L			<0.001^‡^		
No	407 (98.3)	104 (89.7)		1	
Yes	7 (1.7)	12 (10.3)		3.852 (1.350-10.994)	0.012 ^‡^
Anemia			0.086^‡^		
No	340 (82.1)	87 (75.0)			
Yes	74 (17.9)	29 (25.0)			
PLR			0.561		
≤ 92.8	51 (12.3)	12 (10.3)			
> 92.8	363 (87.7)	104 (89.3)			
NLR			0.029^‡^		
≤ 2.75	294 (71.0)	70 (60.3)			
> 2.75	120 (29.0)	46 (39.7)			
CCI			0.001 ^‡^		
0	225 (54.3)	41 (35.3)			
1-2	171 (41.8)	69 (59.5)			
3-6	16 (3.9)	6 (5.2)			
ASA score			0.017 ^‡^		
1-2	350 (84.5)	87 (75.0)			
3-4	64 (15.5)	29 (25.0)			
Hypertension			0.335		
No	311 (75.1)	82 (70.7)			
Yes	103 (24.9)	34 (29.3)			
Diabetes Mellitus			0.297		
No	358 (88.9)	99 (85.3)			
Yes	46 (11.1)	17 (14.7)			
Tumor size, cm			0.001^‡^		
< 4.75	282 (68.1)	60 (51.7)			
≥ 4.75	132 (31.9)	56 (48.3)			
Tumor location			0.318		
Cardia	53 (12.8)	16 (13.8)			
Corpus	85 (20.5)	22 (19.0)			
Pylorus	268 (64.7)	66 (56.9)			
Total	8 (1.9)	12 (10.3)			
Pathological type			0.860		
Non-ulcerative	51 (12.3)	15 (12.9)			
Ulcerative	363 (87.7)	101 (87.1)			
TNM stages			0.001^‡^		
I	145 (35.0)	23 (19.8)			
II	90 (21.7)	25 (21.6)			
III	179 (43.2)	68 (58.6)			
Total gastrectomy			<0.001^‡^		
No	275 (66.4)	54 (46.6)		1	
Yes	139 (33.6)	62 (53.4)		1.916 (1.229-2.988)	0.004 ^‡^
Combined resection			<0.001^‡^		
No	388 (93.7)	96 (82.8)		1	
Yes	26 (6.3)	20 (17.2)		2.231 (1.132-4.398)	0.020 ^‡^
Surgical method			<0.001^‡^		
Open	292 (70.5)	102 (87.9)		1	
Laparoscopic	122 (29.5)	14 (12.1)		0.450 (0.241-0.840)	0.012 ^‡^
Time of Operation, min			0.830		
< 210	223 (53.9)	64 (55.2)			
≥ 210	191 (46.1)	52 (44.8)			
Reconstruction methods			0.005^‡^		
Roux-en-Y	166 (40.1)	68 (58.6)			
Billroth I	174 (42.0)	31 (26.7)			
Billroth II	74 (17.9)	17 (14.7)			
Intraoperative Bleeding			0.028^‡^		
No	359 (86.7)	91 (78.4)			
Yes	55 (13.3)	25 (21.6)			
Intraoperative blood transfusion			<0.001^‡^		
No	398 (96.2)	98 (84.5)		1	
Yes	16 (3.8)	18 (15.5)		2.649 (1.227-5.716)	0.013 ^‡^
Postsugery creatinine change			0.011 ^‡^		
Normal group	173 (41.8)	57 (49.1)		1	
Elevated creatinine group	56 (13.5)	27 (23.2)		1.367 (0.760-2.457)	0.296
Reduced creatinine group	185 (44.7)	32 (27.6)		0.572 (0.345-0.950)	0.031 ^‡^

SPC, severe postoperative complications; BMI, body mass index; PLR, platelet/lymphocyte ratio; NLR, neutrophil/lymphocyte ratio; ASA, American Society of Anesthesiologists; CCI, Charlson comorbidity index; NRS, nutritional risk screening; TNM, tumor-node-metastasis.

Values are number of patients and percent unless indicated otherwise; ^‡^Values are statistically significant (P<0.05).

**Figure 3:**
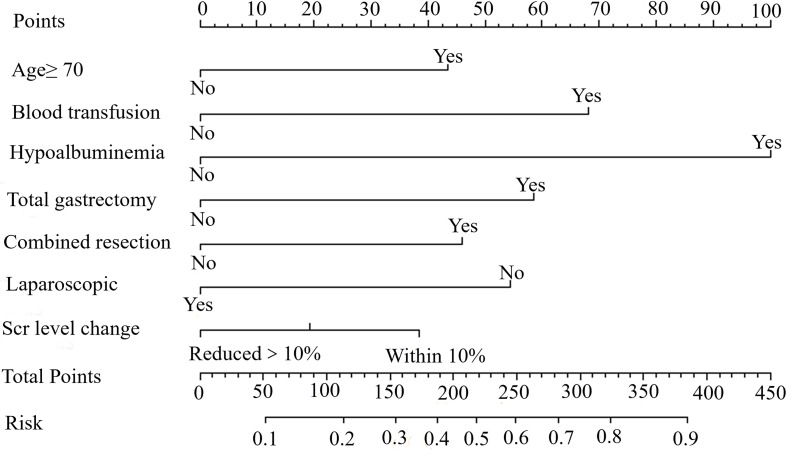
A nomogram indicating the probability of PCs for young GC patients. An example of a nomogram-estimating PCs risk in resected gastric cancer. Draw an upward vertical line from the covariate to the points bar to calculate points. Based on the sum of the covariate points, draw a downward vertical line from the total points line to calculate risk of PCs.

A nomogram based on independent risk factors was constructed to predict the probability of PCs (≥ grade IIa) (**Figure 3**). A discrimination concordance index of 0.715 indicated a 71.5% correct ordering of risk across pairs of patients and a good calibration of observed versus predicted PCs.

### Univariate and multivariate analyses logistic regression analyses for SPCs

The relationship between postsurgical SCr change and SPCs was also evaluated in this study. The univariate analysis results showed that age (P = 0.001), NRS score (P = 0.030), Charlson comorbidity index (P = 0.070), ASA score (P = 0.061), TNM stage (P = 0.037), total gastrectomy (P = 0.007), combined resection (P < 0.001), laparoscopic surgery (P = 0.003), reconstruction methods (P = 0.031), and intraoperative bleeding (P = 0.023) differed significantly (**Table 4**). Variables with a P value < 0.1 on univariate analysis were included in the multivariate logistic regression analysis. In multivariate analysis, age, combined resection, laparoscopic surgery, and postsurgical SCr change, were independently associated with SPCs (all P < 0.05).

**Table 4:** Univariate and multivariate analysis associated with severe postoperative complications.

Factors	Univariate Analysis			Multivariate Analysis	
	Non- SPC (n=475)	SPC (n=55)	P values	OR (95% CI)	P values
Age, years			0.001^‡^		0.008^‡^
< 70	318 (66.9)	24 (43.6)		1	
≥ 70	157 (33.1)	31 (56.4)		2.255 (1.242-4.095)	
Gender			0.592		
Female	114 (24.0)	15 (27.3)			
Male	361 (76.0)	40 (72.7)			
BMI, kg/cm2			0.513		
< 25	389 (81.9)	47 (85.5)			
≥ 25	86 (18.1)	8 (14.5)			
NRS score			0.030^‡^		
1-2	302 (63.6)	30 (54.5)			
3-4	144 (30.3)	16 (29.1)			
5-6	29 (6.1)	9 (16.4)			
Hypoalbuminemia, g/L			0.242		
No	460 (96.8)	51 (92.7)			
Yes	15 (3.2)	4 (7.3)			
Anemia			0.543		
No	381 (80.2)	46 (83.6)			
Yes	94 (19.8)	9 (16.4)			
PLR			0.499		
≤ 92.8	58 (12.2)	5 (9.1)			
> 92.8	517 (87.8)	50 (90.9)			
NLR			0.586		
≤ 2.75	328 (69.1)	36 (65.5)			
> 2.75	147 (30.9)	19 (34.5)			
CCI			0.070 ^‡^		
0	245 (51.6)	21 (38.2)			
1-2	211 (44.4)	31 (56.4)			
3-6	19 (4.0)	3 (5.5)			
ASA score			0.061^‡^		
1-2	393 (82.7)	44 (80.0)			
3-4	82 (17.3)	11 (20.0)			
Hypertension			0.799		
No	353 (74.3)	40 (72.7)			
Yes	122 (25.7)	15 (27.3)			
Diabetes Mellitus			0.279		
No	421 (88.6)	46 (83.6)			
Yes	54 (11.4)	9 (16.4)			
Tumor size, cm			0.181		
< 4.75	311 (65.5)	31 (56.4)			
≥ 4.75	164 (34.5)	24 (43.6)			
Tumor location			0.661		
Cardia	60 (12.6)	9 (16.4)			
Corpus	97 (20.4)	10 (18.2)			
Pylorus	305 (64.2)	29 (52.7)			
Total	13 (2.7)	7 (12.7)			
Pathological type			0.948		
Non-ulcerative	59 (12.4)	7 (12.7)			
Ulcerative	416 (87.6)	48 (87.3)			
TNM stages			0.037^‡^		
I	157 (33.1)	11 (20.0)			
II	103 (21.7)	12 (21.8)			
III	215 (45.3)	32 (58.2)			
Total gastrectomy			0.007^‡^		
No	304 (64.0)	25 (45.5)			
Yes	171 (36.0)	30 (54.5)			
Combined resection			<0.001^‡^		
No	443 (93.3)	41 (74.5)			
Yes	32 (6.7)	14 (25.5)		3.846 (1.830-8.082)	<0.001^‡^
Surgical method			0.003^‡^		
Open	344 (72.4)	50 (90.9)		1	
Laparoscopic	131 (27.6)	5 (9.1)		0.366 (0.139-0.962)	0.042^‡^
Time of Operation, min			0.610		
< 210	259 (54.5)	28 (50.9)			
≥ 210	216 (45.5)	27 (49.1)			
Reconstruction methods			0.031^‡^		
Roux-en-Y	200 (42.1)	34 (61.8)			
Billroth I	192 (40.4)	12 (23.6)			
Billroth II	83 (17.5)	8 (14.5)			
Intraoperative Bleeding			0.023^‡^		
No	409 (86.1)	41 (74.5)			
Yes	66 (13.9)	14 (25.5)			
Intraoperative Blood transfusion			0.009^‡^		
No	449	47			
Yes	28	6			
Postsugery creatinine change			0.001^‡^		
Normal group	208 (43.8)	22 (40.0)		1	
Elevated creatinine group	65 (13.7)	18 (32.7)		2.475 (1.207-5.057)	0.013^‡^
Reduced creatinine group	202 (42.5)	15 (27.3)		0.791 (0.390-1.604)	0.515

SPC, severe postoperative complications; BMI, body mass index; PLR, platelet/lymphocyte ratio; NLR, neutrophil/lymphocyte ratio; ASA, America; Society of Anesthesiologists; CCI, Charlson comorbidity index; NRS, nutritional risk screening; TNM, tumor-node-metastasis.

Values are number of patients and percent unless indicated otherwise; ‡Values are statistically significant (P<0.05).

## Discussion

The incidence of PCs was 21.9% in this study. The median hospital stays in the PC group vs. the non-PC group was 25 vs. 12 days. Additionally, patients with PCs spent significantly more during their hospitalization, which is consistent with the conclusions of previous studies (4–9). The emergence of PCs will prolong the hospital stay, increase personal burden, reduce the trust between doctors and patients, and make it more likely to have conflicts between doctors and patients (20, 21). Therefore, it is particularly important to identify and intervene in patients with complications after radical GC surgery to reduce the occurrence of PCs.

The clinical indicators used to predict the short-term prognosis of patients with GC have their own shortcomings, such as sarcopenia, in assessing the physical function of patients through imaging data, which require physicians to have a certain degree of imaging knowledge (22–24). In contrast, calculating postoperative changes in SCr levels is easier and more efficient, which can be mastered only by short-term learning and more suitable for heavy clinical work. Moreover, the novel predictive model, based on postoperative creatinine changes, age, hypoalbuminemia, total gastrectomy, combined resection, and laparoscopic surgery can be used to assist surgeons in distinguishing low-risk and high-risk patients, ensuring successful perioperative management, and postoperative rehabilitation to reduce the incidence of PCs in high-risk patients (the discriminant consistency index is 0.715).

This is the first time we reported that elevated SCr is an independent risk factor for SPCs (OR = 2.475, P = 0.013) and reduced SCr is an independent protective factor for PCs (OR = 0.559, P = 0.024), which is maintained after including multiple risk indicators in the multivariate analysis. As a large part of the population suffering from GC, elderly patients are more likely to have hemodynamic instability after surgery, which can increase the incidence of renal insufficiency and make patients more susceptible to PCs (25, 26). Latest research shows that minor changes in SCr after surgery are also closely related to the poor prognosis of patients (15, 16). However, most of the research in this area focuses on cardiothoracic surgery research (27–29). No study to our knowledge has found that slight changes in postoperative SCr is related to the short-term prognosis of GC patients underwent curative gastrectomy. Renal insufficiency will trigger the continuous release of systemic pro-inflammatory factors and an immunosuppressive state, making patients more prone to bone loss and malnutrition (30), eventually leading to PCs. Meanwhile, postoperative SCr decrease is likely related to hemodilution, which is considered to be one of the protective factors of kidney function (31, 32), and further related to the decrease in the incidence of PCs in our experiment. However, it is worth noting that this study did not find that elevated SCr is independently associated with PCs of GC. This may be due to our insufficient sample size. In subsequent studies, we should further expand the research volume to explore whether the increase in SCr is related to PCs.

The results in **Table 1** show that there were significant differences in the postoperative hospital stay between the three groups, and the reduced SCr group had significantly lower hospital costs than the other two groups (P < 0.05). The previous data show that the deterioration of renal function is associated with a significantly longer hospital stay, an higher in hospital costs, and an increased risk of death in the hospital (33). Compared with the other two groups, the reduced creatinine group had a lower incidence of pulmonary complications, thrombosis, and intestinal obstruction (**Table 2**). Interventions for these complications will inevitably increase patient costs, but they can return to normal status quickly after appropriate treatment, which may partly explain why there is small difference in postoperative hospital stay between the three groups of patients. We should strengthen early postoperative monitoring of small changes in SCr levels in clinical work, and initiate renal protective interventions to prevent complications after radical GC surgery.

In this study, we also found that age, combined resection, and non-laparoscopic surgery are not only independently related to the occurrence of PCs in patients after radical GC surgery, but also independent risk factors for severe PCs. Elderly patients have poor tolerance to surgery (34), greater surgical trauma after combined organ resection (35), and better exposure to laparoscopic surgery compared to open surgery for surgeon’s operations (36, 37), resulting in differences in the incidence of surgery-related complications. Therefore, for elderly patients with GC, we recommend that under the premise of achieving radical resection, the preoperative choice of endoscopic surgery is performed, and intraoperative resection is as non-combined as possible to reduce the occurrence of PCs. Previous studies have confirmed that GC patients with preoperative hypoalbuminemia are more likely to develop infections and are related to poor prognosis (38). In this study, we found that preoperative hypoalbuminemia was related to PCs, and not an independent risk factor for severe PCs. We speculate that this may be related to the incidence of hypoproteinemia in this experiment is only 3.5%, which is much lower than the 6% reported in previous studies (23).

Several potential limitations still exist in our study and careful consideration is required. This study only explored the relationship between postoperative SCr changes and the short-term prognosis of patients with GC, without the long-term prognosis. In the follow-up work, we will supplement this part of the research. Moreover, the research conclusions of this study come from a single-center subject and lack the support of multi-center data. We will further improve this in the follow-up research.

## Conclusions

This is the first time we reported that minor changes in serum creatinine is an independent risk factor for PCs. Post-surgery, reduced SCr is a protective factor for PCs, while elevated serum creatinine is an independent risk factor for SPCs. Calculating postoperative changes in SCr levels is easier and more efficient, which can be mastered only by short-term learning and more suitable for heavy clinical work. Our nomogram is a simple and practical instrument to identify patients at high risk of surgical complications.

## Data Availability

The raw data supporting the conclusions of this article will be made available by the authors, without undue reservation.

## References

[1] Bray F Ferlay J Soerjomataram I Siegel RL Torre LA Jemal A. Global cancer statistics 2018: GLOBOCAN estimates of incidence and mortality worldwide for 36 cancers in 185 countries. CA Cancer J Clin. (2018) 68:394–424. doi: 10.3322/caac.21492 30207593

[2] Ferlay J Soerjomataram I Dikshit R Eser S Mathers C Rebelo M. Cancer incidence and mortality worldwide: sources, methods and major patterns in GLOBOCAN 2012. Int J Cancer. (2015) 136:E359–86. doi: 10.1002/ijc.29210 25220842

[3] Choi YY Cho M Kwon IG Son T Kim H-I Choi SH. Ten thousand consecutive gastrectomies for gastric cancer: perspectives of a master surgeon. Yonsei Med J. (2019) 60:235–42. doi: 10.3349/ymj.2019.60.3.235 PMC6391520 30799586

[4] Yasunaga H Horiguchi H Kuwabara K Matsuda S Fushimi K Hashimoto H. Outcomes after laparoscopic or open distal gastrectomy for early-stage gastric cancer: a propensity-matched analysis. Ann Surg. (2013) 257:640–6. doi: 10.1097/SLA.0b013e31826fd541 23023204

[5] Yu J Hu J Huang C Ying M Peng X Wei H. The impact of age and comorbidity on postoperative complications in patients with advanced gastric cancer after laparoscopic D2 gastrectomy: results from the Chinese laparoscropic gastrointestinal surgery study (CLASS) group. Eur J Surg Oncol. (2013) 39:1144–9. doi: 10.1016/j.ejso.2013.06.021 23850088

[6] Kim HH Han SU Kim MC Hyung WJ Kim W Lee HJ. Long-term results of laparoscopic gastrectomy for gastric cancer: a large-scale case-control and case-matched Korean multicenter study. J Clin Oncol. (2014) 32:627–33. doi: 10.1200/JCO.2013.48.8551 24470012

[7] Goglia. M Pepe. S Pace. M Fattori. L Minervini. A Giulitti. D. Complication of gastric cancer surgery: A single centre experience. In Vivo (Athens Greece). (2023) 37:2166–72. doi: 10.21873/invivo.13315 PMC10500523 37652505

[8] Tokunaga M Tanizawa Y Bando E Kawamura T Terashima M. Poor survival rate in patients with postoperative intra-abdominal infectious complications following curative gastrectomy for gastric cancer. Ann Surg Oncol. (2013) 20:1575–83. doi: 10.1245/s10434-012-2720-9 23076557

[9] Zhang WT Lin J Chen WS Huang YS Wu RS Chen XD. Sarcopenic obesity is associated with severe postoperative complications in gastric cancer patients undergoing gastrectomy: a prospective study. J Gastrointest Surg. (2018) 22:1861–9. doi: 10.1007/s11605-018-3835-5 29943139

[10] Meersch M Schmidt C Zarbock A. Patient with chronic renal failure undergoing surgery. Curr Opin Anaesthesiol. (2016) 29:413–20. doi: 10.1097/ACO.0000000000000329 26945308

[11] Mooney JF Chow CK Hillis GS. Perioperative renal function and surgical outcome. Curr Opin Anaesthesiol. (2014) 27:195–200. doi: 10.1097/ACO.0000000000000054 24509435

[12] Squires MH 3rd Lad NL Fisher SB Kooby DA Weber SM Brinkman A. The effect of preoperative renal insufficiency on postoperative outcomes after major hepatectomy: a multi-institutional analysis of 1,170 patients. J Am Coll Surg. (2014) 219:914–22. doi: 10.1016/j.jamcollsurg.2014.05.015 25260685

[13] Matsumoto A Nakagawa T Kanatani A Ikeda M Kawai T Miyakawa J. Preoperative chronic kidney disease is predictive of oncological outcome of radical cystectomy for bladder cancer. World J Urol. (2018) 36:249–56. doi: 10.1007/s00345-017-2141-2 29185045

[14] Wang W Wang Y Xu R Chai J Zhou W Chen H. Outcomes following coronary artery bypass graft surgery in patients with mild preoperative renal insufficiency. Braz J Cardiovasc Surg. (2018) 33:155–61. doi: 10.21470/1678-9741-2017-0148 PMC5985842 29898145

[15] Lassnigg A Schmidlin D Mouhieddine M Bachmann LM Druml W Bauer P. Minimal changes of serum creatinine predict prognosis in patients after cardiothoracic surgery: a prospective cohort study. J Am Soc Nephrol. (2004) 15:1597–605. doi: 10.1097/01.ASN.0000130340.93930.DD 15153571

[16] Praught ML Shlipak MG. Are small changes in serum creatinine an important risk factor? Curr Opin Nephrol Hypertens. (2005) 14:265–70. doi: 10.1097/01.mnh.0000165894.90748.72 15821421

[17] Japanese Gastric Cancer Association. Japanese gastric cancer treatment guidelines 2014 (ver. 4). Gastric Cancer. (2017) 20:1–19. doi: 10.1007/s10120-016-0622-4 PMC5215069 27342689

[18] Clavien PA Barkun J de Oliveira ML Vauthey JN Dindo D Schulick RD. The Clavien-Dindo classification of surgical complications: five-year experience. Ann Surg. (2009) 250:187–96. doi: 10.1097/SLA.0b013e3181b13ca2 19638912

[19] Balachandran VP Gonen M Smith JJ DeMatteo RP. Nomograms in oncology: more than meets the eye. Lancet Oncol. (2015) 16:e173–80. doi: 10.1016/S1470-2045(14)71116-7 PMC4465353 25846097

[20] Wang S Xu L Wang Q Li J Bai B Li Z. Postoperative complications and prognosis after radical gastrectomy for gastric cancer: a systematic review and meta-analysis of observational studies. World J Surg Oncol. (2019) 17:52. doi: 10.1186/s12957-019-1593-9 30885211 PMC6423865

[21] Blitz JD Shoham MH Fang Y Narine V Mehta N Sharma BS. Preoperative renal insufficiency: underreporting and association with readmission and major postoperative morbidity in an academic medical center. Anesth analgesia. (2016) 123:1500–15. doi: 10.1213/ANE.0000000000001573 27861446

[22] Zhuang CL Huang DD Pang WY Zhou CJ Wang SL Lou N. Sarcopenia is an independent predictor of severe postoperative complications and long-term survival after radical gastrectomy for gastric cancer: analysis from a large-scale cohort. Med (Baltimore). (2016) 95:e3164. doi: 10.1097/MD.0000000000003164 PMC4998538 27043677

[23] Shi B Liu S Chen J Liu J Luo Y Long L. Sarcopenia is associated with perioperative outcomes in gastric cancer patients undergoing gastrectomy. Ann Nutr Metab. (2019) 75:213–22. doi: 10.1159/000504283 31846973

[24] Ongaro E Buoro V Cinausero M Caccialanza R Turri A Fanotto V. Sarcopenia in gastric cancer: when the loss costs too much. Gastric Cancer. (2017) 20:563–72. doi: 10.1007/s10120-017-0722-9 28477106

[25] Prowle JR Kam EP Ahmad T Smith NC Protopapa K Pearse RM. Preoperative renal dysfunction and mortality after non-cardiac surgery. Br J Surg. (2016) 103:1316–25. doi: 10.1002/bjs.10186 27346181

[26] Weiss R Meersch M Pavenstädt HJ Zarbock A. Acute kidney injury: A frequently underestimated problem in perioperative medicine. Deutsches Arzteblatt Int. (2019) 116:833–42. doi: 10.3238/arztebl.2019.0833 PMC6962766 31888797

[27] Husain-Syed F Ferrari F Sharma A Hinna Danesi T Bezerra P Lopez-Giacoman S. Persistent decrease of renal functional reserve in patients after cardiac surgery-associated acute kidney injury despite clinical recovery. Nephrol Dial Transplant. (2019) 34:308–17. doi: 10.1093/ndt/gfy227 30053231

[28] Xu J Yu J Xu X Shen B Wang Y Jiang W. Preoperative hidden renal dysfunction add an age dependent risk of progressive chronic kidney disease after cardiac surgery. J cardiothoracic Surg. (2019) 14:151. doi: 10.1186/s13019-019-0977-9 PMC6704689 31438993

[29] Dardashti A Ederoth P Algotsson L Brondén B Bjursten H. Incidence, dynamics, and prognostic value of acute kidney injury for death after cardiac surgery. J Thorac Cardiovasc Surg. (2014) 147:800–7. doi: 10.1016/j.jtcvs.2013.07.073 24100099

[30] Kurts C Panzer U Anders HJ Rees AJ. The immune system and kidney disease: basic concepts and clinical implications. Nat Rev Immunol. (2013) 13:738–53. doi: 10.1038/nri3523 24037418

[31] Rajagopalan PR Reines HD Pulliam C Fitts CT LeVeen HH. Reversal of acute renal failure using hemodilution with hydroxyethyl starch. J Trauma. (1983) 23:795–800. doi: 10.1097/00005373-198309000-00004 6194306

[32] Swaminathan M Phillips-Bute BG Conlon PJ Smith PK Newman MF Stafford-Smith M. The association of lowest hematocrit during cardiopulmonary bypass with acute renal injury after coronary artery bypass surgery. Ann Thorac Surg. (2003) 76:784–91;discussion 792. doi: 10.1016/S0003-4975(03)00558-7 12963200

[33] Krumholz HM Chen YT Vaccarino V Wang Y Radford MJ Bradford WD. Correlates and impact on outcomes of worsening renal function in patients > or =65 years of age with heart failure. Am J Cardiol. (2000) 85:1110–3. doi: 10.1016/S0002-9149(00)00705-0 10781761

[34] Fujisaki M Shinohara T Hanyu N Kawano S Tanaka Y Watanabe A. Laparoscopic gastrectomy for gastric cancer in the elderly patients. Surg Endosc. (2016) 30:1380–7. doi: 10.1007/s00464-015-4340-5 26123337

[35] Huang YS Chen XD Shi MM Xu LB Wang SJ Chen WS. Diffuse reduction of spleen density is an independent predictor of post-operative outcomes after curative gastrectomy in gastric cancer: A multi-center study. Front Oncol. (2020) 10:1050. doi: 10.3389/fonc.2020.01050 32714867 PMC7340088

[36] Tsekrekos A Klevebro F Hayami M Kamiya S Lindblad M Nilsson M. Laparoscopic versus open gastrectomy for cancer: A western center cohort study. J Surg Res. (2020) 247:372–9. doi: 10.1016/j.jss.2019.10.006 31679797

[37] Honda M Kumamaru H Etoh T Miyata H Yamashita Y Yoshida K. Surgical risk and benefits of laparoscopic surgery for elderly patients with gastric cancer: a multicenter prospective cohort study. Gastric Cancer. (2019) 22:845–52. doi: 10.1007/s10120-018-0898-7 30539321

[38] Liu ZJ Ge XL Ai SC Wang HK Sun F Chen L. Postoperative decrease of serum albumin predicts short-term complications in patients undergoing gastric cancer resection. World J Gastroenterol. (2017) 23:4978–85. doi: 10.3748/wjg.v23.i27.4978 PMC5526768 28785152

